# Perceptual and Conceptual Distortions of Implicit Hand Maps

**DOI:** 10.3389/fnhum.2015.00656

**Published:** 2015-12-16

**Authors:** Matthew R. Longo, Stefania Mattioni, Nataşa Ganea

**Affiliations:** ^1^Department of Psychological Sciences, Birkbeck University of LondonLondon, UK; ^2^Centre for Mind/Brain Sciences, University of TrentoTrento, Italy

**Keywords:** body representation, position sense, perceptual distortions, somatosensation

## Abstract

Recent studies have revealed that human position sense relies on a massively distorted representation of hand size and shape. By comparing the judged location of landmarks on an occluded hand, Longo and Haggard (2010) constructed implicit perceptual maps of represented hand structure, showing large underestimation of finger length and overestimation of hand width. Here, we investigated the contribution of two potential sources of distortions to such effects: *perceptual distortions* reflecting spatial warping of the representation of bodily tissue itself, perhaps reflecting distortions of somatotopic cortical maps, and *conceptual distortions* reflecting mistaken beliefs about the locations of different landmarks within the body. In Experiment 1 we compared distorted hand maps to a task in which participants explicitly judged the location of their knuckles in a hand silhouette. The results revealed the conceptual distortions are responsible for at least part of the underestimation of finger length, but cannot explain overestimation of hand width. Experiment 2 compared distortions of the participant’s own hand based on position sense with a prosthetic hand based on visual memory. Underestimation of finger length was found for both hands, providing further evidence that it reflects a conceptual distortion. In contrast, overestimation of hand width was specific to representation of the participant’s own hand, confirming it reflects a perceptual distortion. Together, these results suggest that distorted body representations do not reflect a single underlying cause. Rather, both perceptual and conceptual distortions contribute to the overall configuration of the hand representation.

## Introduction

Distorted body representations and delusional beliefs about the body are a conspicuous feature of many neurological and psychiatric disorders. Such misperceptions have received widespread interest both for their clinical importance and because of their strikingly bizarre nature, which stands in stark contrast to the intimate knowledge we seem to have about our own body. Some examples of such conditions include: phantom limbs in which a limb which has been amputated is perceived as continuing to exist (Melzack, 1992; Ramachandran and Hirstein, 1998), somatoparaphrenia in which the patient denies that their limb actually belongs to them (Vallar and Ronchi, 2009), asomatagnosia in which one half of the body is perceived as being absent (Critchley, 1953), delusions in eating disorders in which emaciated patients claim to be fat (Treasure et al., 2010), body integrity identity disorder (or xenomelia) in which people express the desire to have some part of their body amputated (First, 2005; Brugger et al., 2013), and autoscopic illusions and out-of-body experiences in which people experience a decoupling of the location of their body and their first-person perspective (Brugger et al., 1997; Blanke et al., 2004).

### Distortions of Body Representations in Healthy Adults

A growing body of recent research has begun to show that such distortions are not specific to disease, but that healthy adults show striking misrepresentations of their body. For example, our own recent research has investigated representations of body size and shape underlying position sense. While several forms of proprioceptive afferent signal provide information about body posture in terms of the degree of flexion or extension of each joint (Proske and Gandevia, 2012), in order to perceive the absolute position in space of one part of the body such information about joint angles needs to be combined with information about the length of bodily segments between joints which is not specified by any immediate afferent signal (Longo et al., 2010). Longo and Haggard (2010) developed a method for isolating and measuring this body representation, what they called the *body model*, in the specific case of the hand. Participants laid their hand flat on a table and used a long baton to indicate the perceived location of landmarks on their hand (i.e., knuckles and fingertips) on an occluding board. By comparing the relative locations of the judgments of different landmarks, Longo and Haggard (2010) constructed perceptual maps of hand structure, which could then be compared to the actual hand structure. Remarkably, these maps were massively distorted, in a highly stereotyped way across people. Specifically, there were three distinct distortions that appeared consistently: (1) an overall overestimation of hand width, (2) an overall underestimation of finger length, and (3) a radial-ulnar gradient with underestimation increasing from the thumb to little finger.

This overall pattern of distortions to the body model has now been replicated several times, both by us (Longo and Haggard, 2012a,b; Longo et al., 2012; Longo, 2014; Mattioni and Longo, 2014) and by others (Lopez et al., 2012; Ferrè et al., 2013; Saulton et al., 2015). Similar distortions have been found for the left and right hands (Longo and Haggard, 2010, Experiment 3), when the hand is rotated relative to the rest of the body (Longo and Haggard, 2010, Experiment 2), when participants respond with vision or while blindfolded (Longo, 2014), whether landmarks are cued using verbal labels or touch (Mattioni and Longo, 2014), and on the dorsal and palmar hand surfaces (Longo and Haggard, 2012a), though their magnitude is reduced on the palm. Other studies have described analogous distortions across wider regions of the body surface (e.g., Cardinali et al., 2009, 2011; Hach and Schütz-Bosbach, 2010; Hach et al., 2011).

While the present paper focuses on these distortions of body representations underlying proprioception, it is important to note that other studies have revealed distortions across a range of other tasks reflecting various perceptual and cognitive abilities (for a recent review see Longo, 2015b). For example, studies of tactile distance perception have shown large distortions across different skin surfaces (e.g., Weber, 1834/1996; Cholewiak, 1999; Taylor-Clarke et al., 2004; de Vignemont et al., 2005; Anema et al., 2008), as a function of orientation within a single surface (e.g., Green, 1982; Longo and Haggard, 2011; Canzoneri et al., 2013; Longo and Sadibolova, 2013; Miller et al., 2014), and across body-part boundaries (e.g., de Vignemont et al., 2009; Le Cornu Knight et al., 2014). Other studies have described distortions underlying localisation of tactile, thermal, and nociceptive stimuli on the skin surface (e.g., Trojan et al., 2006; Mancini et al., 2011; Steenbergen et al., 2012; Margolis and Longo, 2015). Finally, some recent studies have also found distortions for more explicit tasks involving judgments of the relative location of different body landmarks (e.g., Fuentes et al., 2013a,b,c) and the length of different body parts (e.g., Linkenauger et al., 2009, 2015; Longo and Haggard, 2012b). Thus, far from being specific to disease, distorted body representations appear to be a widespread characteristic of healthy mental life.

### Perceptual vs. Conceptual Interpretations of Distortions

While the experimental evidence for distorted body representations is substantial, the proper interpretation of these effects remains uncertain. In their first experiment using the ‘pointing’ task, Longo and Haggard (2010) compared the distorted implicit hand map with another task involving more explicit judgments of hand shape, based on the template matching procedure introduced by Gandevia and Phegan (1999). Participants were shown arrays of hand images reflecting different stretches applied to a single image of the back of the hand, resulting in a range of hand shapes from extremely slender to extremely wide. They were asked to select from this array the hand image most like the perceived shape of their own hand. In stark contrast to the squat and fat hand maps emerging from the pointing task, judgments in the template matching task were approximately veridical on average. Subsequent studies using similar tasks have also found no evidence for systematic distortions (Longo and Haggard, 2012b; Longo, 2015c). On the basis of this dissociation, Longo and Haggard (2010) suggested that position sense relies on a distorted implicit body representation, distinct from the conscious body image, which (as seen in the template matching task) appears to be largely undistorted.

Longo and Haggard (2010) suggested that the distortions observed in the hand maps from the pointing task could reflect the retention of distortions characteristic of early somatosensory maps, such as the well-known ‘Penfield homunculus’ (Penfield and Boldrey, 1937). Evidence consistent with this interpretation comes from several analogies between the pattern of distortions and known properties of early somatosensory organization. For example, the radial-ulnar gradient of underestimation of finger length mirrors both the relative tactile spatial acuity of the fingers (Vega-Bermudez and Johnson, 2001; Duncan and Boynton, 2007) and their cortical magnification (Duncan and Boynton, 2007), both of which are highest on the thumb and progressively smaller across the hand toward the little finger.

Further, the overestimation of hand width relative to length found in the pointing task mirrors the bias (also described above) to perceive tactile distances as bigger when oriented across the width of the body than along its length (e.g., Green, 1982; Longo and Haggard, 2011). These distortions, furthermore, are reduced on the palmar compared to the dorsal hand surface both for the pointing task (Longo and Haggard, 2012a) and for perceived tactile distance (Longo and Haggard, 2011). Further, both of these perceptual distortions appear to reflect still more basic aspects of somatosensory organization, including the fact that tactile spatial acuity is higher across the width of the body than along its length (e.g., Weber, 1834/1996; Cody et al., 2008) and the fact that the receptive fields of somatosensory neurons in the spinal cord, thalamus, and cortex representing the limbs tend to be oval-shaped, rather than circular, with their long axes running along the proximo-distal axis of the limb (e.g., Brooks et al., 1961; Brown et al., 1975; Alloway et al., 1989).

On the interpretation of Longo and Haggard (2010), position sense relies on a representation of the metric properties of the body that is influenced by more basic somatosensory maps, and retains the distortions of such maps in vestigial form. The relative proportions of this representation then, are stretched in ways that reflect the relative sensitivity of different skin regions, but which have no analog in our high-level cognitive understanding of our body, nor in our subjective body image. People do not believe that their hand is squat and fat, nor do they consciously experience it as such. It was for this reason that Longo and Haggard (2010) referred to the body model as an ‘implicit body representation,’ to emphasize its separation from wider aspects of cognition. On this view, the distortions are purely *perceptual*, both in that they arise from distortions of sensory maps and in that they affect perceptual judgments of bodily location, without interacting with cognition more widely.

Is it in fact the case that these distortions do not affect cognition more broadly? Three recent findings have suggested, in contrast to this suggestion, that distortions may be more widespread than suggested by Longo and Haggard (2010). First, as mentioned above, in a subsequent study Longo and Haggard (2012b) found distortions qualitatively similar to those found in the pointing task using a task in which participants judged whether lines presented on a monitor were shorter or longer than the perceived length of different parts of the hand. Specifically, participants underestimated the length of their fingers (i.e., the distance between the knuckle and tip) and this bias increased from the thumb to the little finger. These distortions were similar to those found in the pointing task, though smaller in magnitude. Because the judgment in this ‘line length’ task is much more explicitly about perceived body size than the pointing task, this pattern suggests the distortions may not be limited to purely implicit tasks.

Second, Saulton et al. (2015) recently showed that distortions analogous to those described by Longo and Haggard (2010) can also be found when participants indicate the remembered location of different landmarks on inanimate objects. For example, when participants were shown objects including a metal rake, a rectangular post-it pad, and a squared box, and then asked to judge the location of different landmarks on each object, their location judgments underestimated object length. While these distortions were again smaller in magnitude than those found for proprioceptive localisation of landmarks on the participant’s own hand, their similar direction again suggests the operation of a more general process affecting localisation based on visual memory as well as on proprioception.

Finally, recent results have suggested that people have genuine conceptual misunderstanding about the location of their knuckles within the hand. Longo (2015a) asked participants to judge the location of their knuckles (i.e., the metacarpophalangeal joint) by positioning the tip of a baton on their palm. Remarkably, participants judged their knuckles as being farther forward in the hand than they actually are for all fingers other than the thumb. Margolis and Longo (2015) showed a similar result using a task in which participants localized their knuckles by clicking the mouse cursor within a silhouette images of their hand. Numerous authors have emphasized the importance of joints for basic aspects of perception (e.g., Cholewiak and Collins, 2003; de Vignemont et al., 2009; Le Cornu Knight et al., 2014) and for providing spatial structure of bodily experience (Bermúdez, 1998). Bermúdez (1998), for example, emphasizes the importance of the joints being “hinges” which provide an obvious segmentation of the body into discrete parts. Nevertheless, people seem to believe that their knuckles are substantially more distal than they actually are.

**FIGURE 1:**
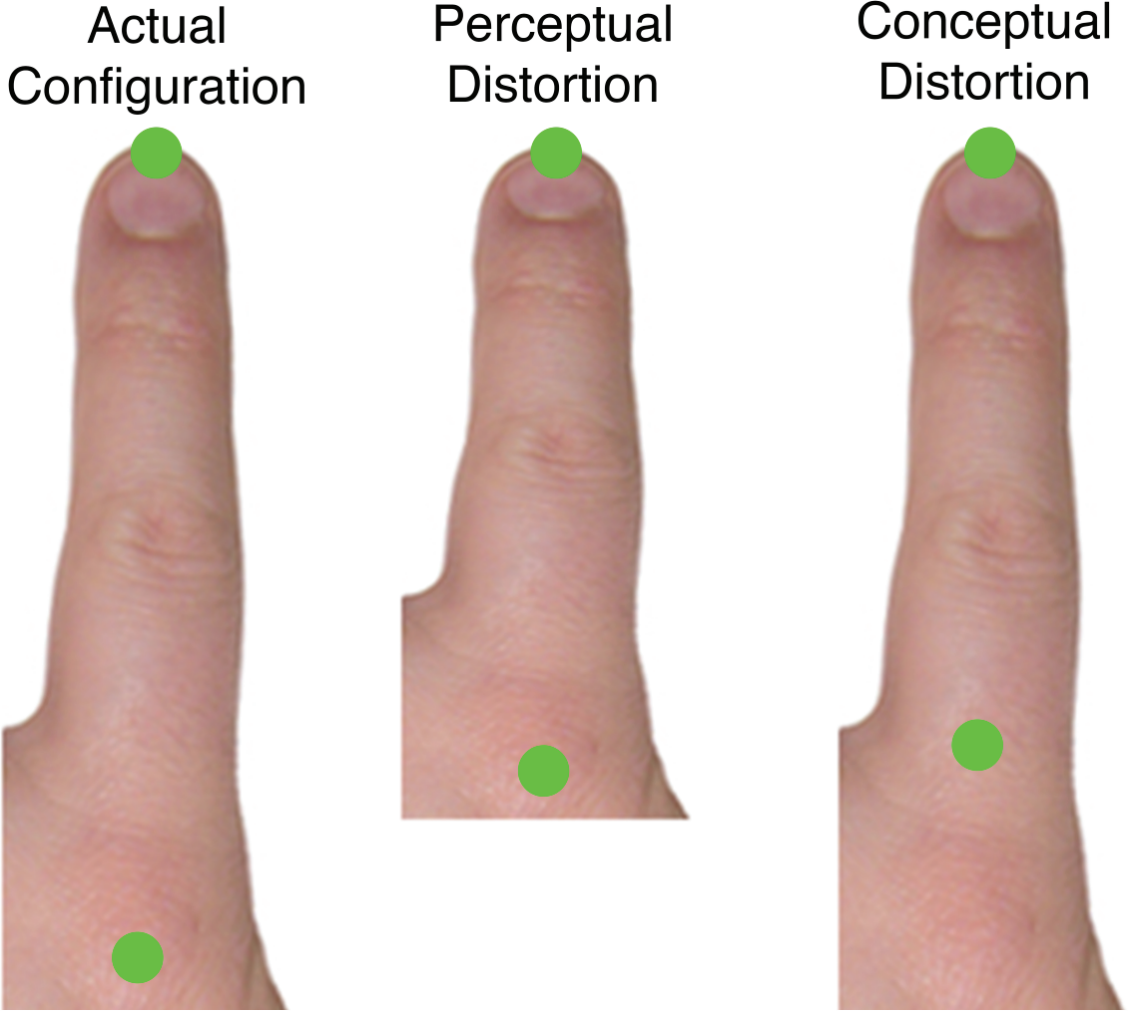
**Two ways in which the distance between landmarks could be distorted.** The **(left)** shows the actual locations of the knuckle and tip of the index finger. The **(center)** panel shows a perceptual distortion in which the representation of the tissue between the landmarks is compressed. The **(right)**, in contrast, shows a conceptual distortion in which participants believe that the knuckle is farther forward in the hand than it actually is.

This last result provides a possible way to reinterpret the distortions found using the localisation task. Misestimation of the distance between landmarks could arise in two quite different ways, either (1) through perceptual stretch of the representation of the tissue between the two landmarks (i.e., *perceptual distortion*), or (2) through mislocalisation of the landmarks themselves within a veridically represented body part (i.e., *conceptual distortion*). **Figure 1** illustrates these two possibilities, which are not mutually exclusive and could both contribute to the distortions observed in previous studies (e.g., Longo and Haggard, 2010).

### The Present Study

This study investigated the contribution of perceptual and conceptual contributions to the distortions observed in body representations underlying position sense (e.g., Longo and Haggard, 2010). In Experiment 1, we compared the magnitude of distortions in the pointing task (Longo and Haggard, 2010) with those in the line length task (Longo and Haggard, 2012b) and the knuckle localisation task (Longo, 2015a; Margolis and Longo, 2015). If the underestimation of finger length in the pointing and line length tasks reflects a genuine belief by participants that their knuckles are farther forward in their hands than they actually are, then the magnitude of distortion should be similar across tasks, and consistent individual differences should be found in the magnitude of distortions across people. To anticipate our results, we do find a significant correlation between distortion in the pointing and knuckle localisation tasks, suggesting that this distortion is, at least partly, conceptual in nature. The magnitude of distortion in the knuckle localisation task, however, was only about one quarter of that in the pointing task. Further, no overestimation of spacing between knuckles was apparent in the knuckle localisation task, suggesting that the conceptual distortion is insufficient to fully account for the perceptual distortions.

In Experiment 2, we investigated this issue by comparing proprioceptive localisation of the participant’s own hand with localisation based on visual memory of landmarks on a seen prosthetic rubber hand. To the extent that distortions reflect conceptual misunderstanding of the organization of hands in general, similar distortions should be found for the participant’s own hand and for the rubber hand. In contrast, to the extent that distortions reflect perceptual distortion of represented hand shape, they should be specific to the participant’s own hand. To anticipate our results, we find that underestimation of finger length appears with similar magnitude for both hands, suggesting that it reflects a largely conceptual distortion, while overestimation of hand width is specific to the participant’s own hand, suggesting it reflects a largely perceptual distortion.

## Experiment 1

**FIGURE 2:**
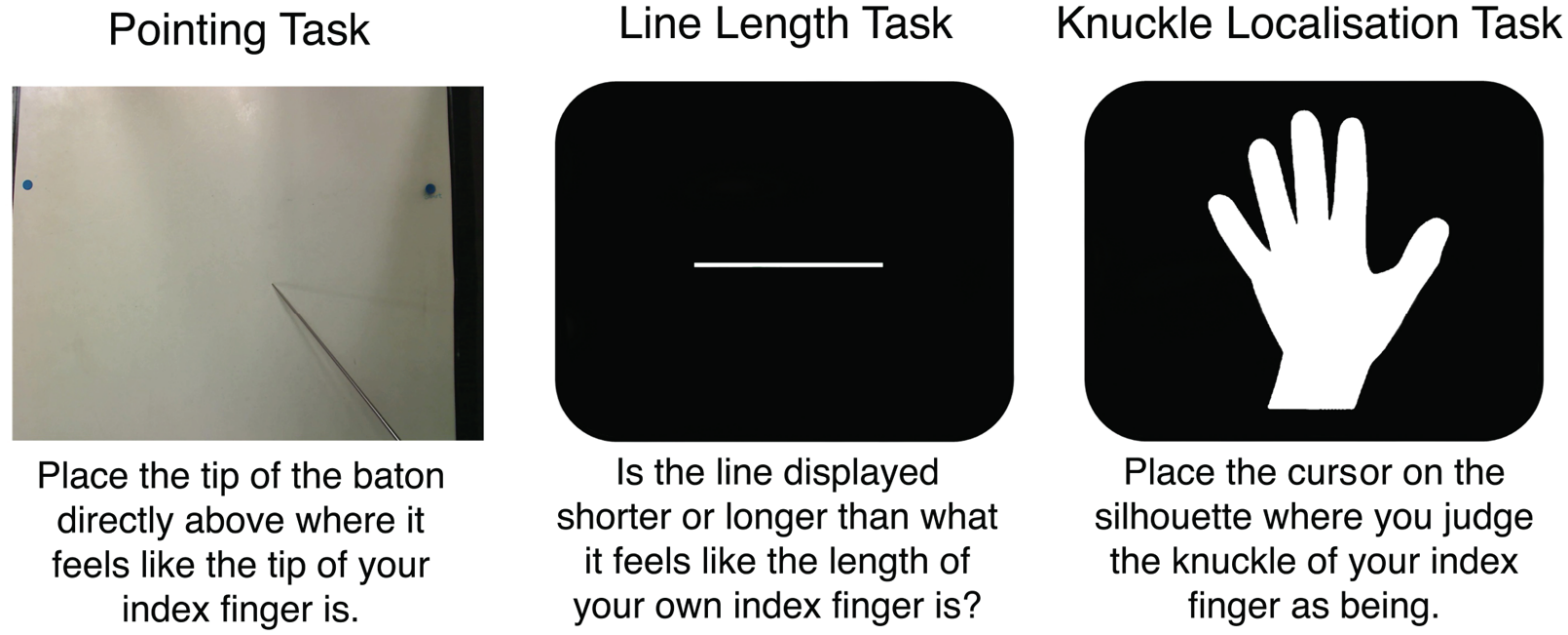
**The three tasks in Experiment 1**.

This experiment compared the magnitude of distortions in three tasks which have been recently shown to produce clear distortions: the pointing task measuring body representations underlying position sense (Longo and Haggard, 2010), the line length task measuring explicit judgments of body-part size (Longo and Haggard, 2012b), and the knuckle localisation task measuring overt beliefs about knuckle location within the hand (Margolis and Longo, 2015). These tasks are shown in **Figure 2**.

### Methods

#### Participants

Twenty healthy individuals (11 females) between 20 and 62 years of age (*M*: 30.9 years). All were right-handed as assessed by the Edinburgh Inventory (Oldfield, 1971; *M*: 83.6; range: 55–100). Procedures were approved by the local ethics committee and were in accordance with the principles of the Declaration of Helsinki.

#### Procedures

There were three tasks administered in a fixed sequence: pointing, line length, knuckle localisation. A fixed order was used in order to keep procedures as consistent as possible across participants to maximize our ability to detect shared individual differences across tasks.

##### Pointing task

The procedure for the pointing task was similar to our previous studies using this paradigm (e.g., Longo and Haggard, 2010, 2012a; Longo, 2014, 2015d; Mattioni and Longo, 2014; Longo et al., 2015b). Participants placed their left palm-down on a table, aligned with their body midline. An occluding board (40 cm × 40 cm) was placed over the hand, resting on four pillars (6 cm high). A camera (Logitech Webcam Pro 9000 HD) suspended on a tripod above the occluding board (27 cm high) captured photographs (1600 pixels × 1200 pixels) controlled by a custom MATLAB (Mathworks, Natick, MA, USA) script.

Participants used a long baton (35 cm length; 2 mm diameter) to indicate with their right hand the perceived location of several landmarks on their occluded left hand. Ten landmarks were used: the knuckles at the base of each finger and the tip of each finger. They were instructed to be precise in their judgments and avoid ballistic pointing or strategies such as tracing the outline of the hand. At the beginning of each trial, the experimenter gave the participant a verbal instruction about which landmark to localize. To ensure that participants understood which landmarks to localize, the researcher labeled and showed on her own hand the tip and the knuckle of each finger at the beginning of the study. To make sure that they judged each landmark individually, participants moved the baton to a yellow dot at the edge of the board before the start of each trial. When the participants indicated their response, a photograph was taken and saved for oﬄine coding.

There were two blocks of 50 trials. Each block included five mini-blocks of one trial of each landmark in random order. At the beginning and the end of each block a photograph of the participant’s hand was taken to measure the true hand proportions and to check that the hand hadn’t moved during the course of the block. To facilitate coding, a black mark was made on the center of each knuckle with a non-permanent felt pen. A 10 cm ruler appeared in the photographs of the participant’s hand and allowed conversion between pixel units and centimeters.

The analysis was similar to our previous studies with this paradigm. The *x*–*y* pixel coordinates of each landmark on the images of actual hands and of all responses were coded using a custom MATLAB script. Mean coordinates were then calculated for each landmark in each experimental block. The set of mean coordinates in each block comprises two maps, one reflecting actual hand shape, the other reflecting represented hand shape. Distances between mean pixel coordinates of the tip and knuckle of each finger and between pairs of knuckles were calculated and converted into cm.

##### Line length task

Procedures for the line length task were similar to those used by Longo and Haggard (2012b). Participants judged whether a line visually presented on a monitor was shorter or longer than the perceived size of parts of their left hand: the length of each of the five fingers and the distance between the knuckles of the index and little fingers. Stimuli were presented by a custom MATLAB script. Viewing distance was approximately 40 cm. There were twelve blocks, two of each body part. We used a staircase procedure (Cornsweet, 1962) to estimate the perceived length of each body part. On each block, the length of a single body part was estimated using four randomly interleaved staircases forming a factorial manipulation of line orientation (horizontal/vertical) and starting size (small: 30 pixels/1.26 cm; large: 500 pixels/21.00 cm). The lines were approximately 2 mm wide and were white on a black background. The initial step size was 2.69 cm (64 pixels). After each reversal the step size was halved. Each staircase ended after five reversals. On each trial, the stimulus was selected randomly from the remaining active staircases. Blocks finished when all staircases had finished. Participants responded by pressing one of two buttons on a keypad with their right hand. Responses were unspeeded. Both of the participant’s hands remained on the lap out of view throughout the experiment.

##### Knuckle localisation task

Procedures for the knuckle localisation task were similar to those of Margolis and Longo (2015). At the start of the experiment, a photograph of the back of the participant’s left hand against a black background was taken with the camera used for the pointing task. This image was cropped and edited using the Threshold tool in the GNU Image Manipulation Program (version 2.8.2) to produce a white silhouette on a black background (600 pixels × 600 pixels/ 25.20 cm × 25.20 cm). During the task, the silhouette was shown continuously under control of a custom MATLAB script. On each trial, the experimenter gave the participant a verbal instruction about which one of their knuckles to localize. The participant had to click the mouse cursor (a thin cross) on the silhouette. There were 16 blocks of 5 trials, each including one trial of each finger in random order. After each response, the mouse cursor appeared at a random location on the monitor.

The actual location of the knuckles and judgments were converted into a common frame of reference using *Bookstein Coordinates* (Bookstein, 1991; Zelditch et al., 2004). To calculate Bookstein coordinates, two landmarks are defined as the locations of coordinates (0,0) and (1,0) and the other landmarks positioned accordingly. As in previous studies with this and similar paradigms (Mancini et al., 2011; Margolis and Longo, 2015), the knuckle of the little finger was defined as point (0,0) and the knuckle of the index finger as point (1,0). This results in the Bookstein *x*-axis being aligned along the medio-lateral hand axis, and the *y*-axis along the proximo-distal axis. Thus, distal localisation bias can be calculated as the difference in Bookstein *y*-coordinates between judged and actual knuckle location. Similarly, the judged and actual distances between pairs of knuckles and between the knuckle and tip of each finger can be calculated.

### Results

#### Pointing Task

**FIGURE 3:**
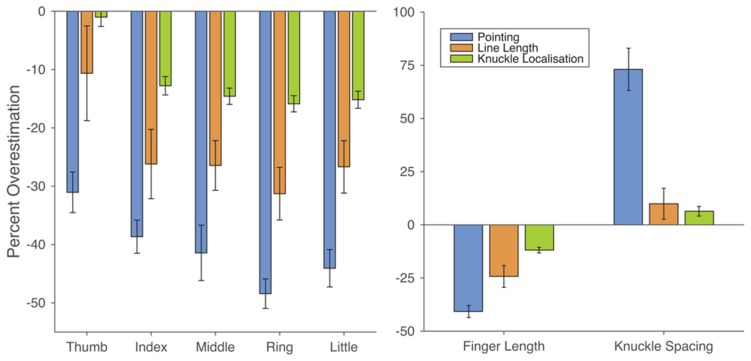
**Results from the three tasks of Experiment 1.** **(Left)** Overestimation of finger length as a function of the five fingers across the three tasks. Clear underestimation was found for all three tasks, which increased across the hand from thumb to little finger. The magnitude of underestimation, however, differed across tasks, being largest for the pointing task, intermediate for the line length task, and smallest for the knuckle localisation task. **(Right)** Overestimation of finger length (i.e., the data from the left panel collapsed across the five fingers) and of hand width (i.e., the distance between the knuckles of the index and little fingers) across the three tasks. While modest overestimation of hand width was found for the line length and knuckle localisation tasks, it was dramatically smaller than in the pointing task.

Across fingers, there was clear underestimation of finger length (*M*: 40.73% underestimation), *t*(19) = 14.55, *p* < 0.0001, *d* = 3.25 (**Figure 3**). The change in magnitude of underestimation of finger length across the hand was quantified using least-squares regression, regressing underestimation for each participant on finger number (i.e., thumb = 1, little finger = 5). Underestimation increased from the thumb to the little finger (mean β = -3.58%/finger), *t*(19) = -6.44, *p* < 0.0001, *d* = 1.44. There was also clear overestimation of hand width. Taking the distance between the knuckles of the index and little fingers as an overall measure of hand width, there was clear overestimation (*M*: 73.1%), *t*(19) = 7.36, *p* < 0.0001, *d* = 1.65. These results provide a clear replication of the characteristic distortions found in previous studies using this paradigm (e.g., Longo and Haggard, 2010, 2012a; Longo, 2014). Moreover, the magnitudes of overestimation of finger length and overestimation of hand width are comparable to those found in previous studies.

#### Line Length Task

Across fingers, there was clear underestimation of finger length (*M*: 24.24%), *t*(19) = -4.74, *p* < 0.0001, *d* = 1.06 (**Figure 3**). The magnitude of underestimation increased from the thumb to little finger (mean β = 3.72%/finger), *t*(19) = -3.29, *p* < 0.005, *d* = 0.74. Unlike the pointing task, however, there was no significant overestimation of hand width (*M*: 9.91%), *t*(19) = 1.36, *p* = 0.190, *d* = 0.30. These results replicate the effects reported by Longo and Haggard (2012b).

#### Knuckle Localisation Task

Across fingers, there was a clear distal bias (*M*: 0.20 Bookstein units), *t*(19) = 9.77, *p* < 0.0001, *d* = 2.18. This clearly replicates the results of Margolis and Longo (2015). This distal bias can be interpreted as a form of underestimation of finger length on the reasonable assumption that participants would correctly localize the fingertips on the silhouettes. Expressed this way there was clear underestimation of finger length (*M*: 11.88%), *t*(19) = 8.98, *p* < 0.0001, *d* = 2.01 (**Figure 3**). As with the other tasks, the magnitude of this bias increased from the thumb to little finger (mean β = 3.14%/finger), *t*(19) = 11.72, *p* < 0.0001, *d* = 2.62. There was also modest, but significant overestimation of the distance between the knuckles of the index and little fingers (*M*: 6.39%), *t*(19) = 2.82, *p* = 0.011, *d* = 0.63.

#### Comparison of Tasks

Clear underestimation of finger length was found in each of the three tasks. To compare the magnitude of distortions across tasks, the overestimation scores were entered into a 3 × 5 ANOVA with *task* (pointing, line length, knuckle localisation) and *finger* (thumb, index, middle, ring, little) as within-subject factors. Unsurprisingly, given the gradient observed in each task individually, there was a significant main effect of finger, *F*(2.25,42.73) = 24.23, *p* < 0.0001, $\eta_{p}^{2}$ = 0.56. More critically, there was a significant effect of task, *F*(1.25,23.71) = 18.32, *p* < 0.0005, $\eta_{p}^{2}$ = 0.49. Collapsing across fingers, underestimation in the proprioceptive localisation task was significantly larger than in the line length, *t*(19) = 2.81, *p* < 0.02, *d*_z_ = 0.63, and knuckle localisation, *t*(19) = 12.42, *p* < 0.0001, *d*_z_ = 2.78, tasks, while underestimation was larger in the line length than the knuckle localisation task, *t*(19) = 2.30, *p* < 0.05, *d*_z_ = 0.51. There was no interaction between task and finger, *F*(3.87,73.55) = 0.933, *p* = 0.447, $\eta_{p}^{2}$ = 0.05, suggesting that the gradient across fingers was similar across the three tasks.

**FIGURE 4:**
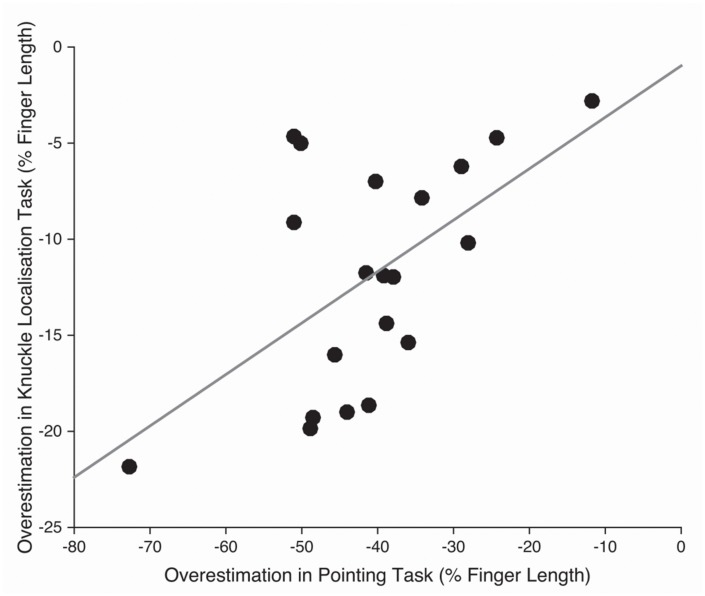
**Scatterplot showing the relation between overestimation of finger length (collapsed across the five fingers) for the pointing and knuckle localisation tasks**.

Despite the differences in magnitude across tasks, there was nevertheless a moderate correlation between the magnitude of underestimation of finger length in the pointing and knuckle localisation tasks, *r*(18) = 0.567, *p* < 0.01 (**Figure 4**). In contrast, despite the underestimation observed in line length task which was qualitatively similar to the other tasks, distortion in the line length task was uncorrelated with distortion in either the pointing, *r*(18) = 0.020, *p* > 0.90, or knuckle localisation, *r*(18) = -0.316, *p* = 0.175, tasks.

Finally, to compare the magnitude of the overestimation of hand width across tasks, a one-way ANOVA was performed. There was a clear effect of task, *F*(2,38) = 41.10, *p* < 0.0001, $\eta_{p}^{2}$ = 0.684. As is clear from **Figure 3**, overestimation was dramatically larger in the pointing task than in either the line length, *t*(19) = 8.67, *p* < 0.0001, *d*_z_ = 1.94, and knuckle localisation, *t*(19) = 6.58, *p* < 0.0001, *d*_z_ = 1.47, tasks. The magnitude of overestimation did not differ between the line length and knuckle localisation tasks, *t*(19) = 0.47, *p* = 0.644, *d*_z_ = 0.106. In contrast to the significant correlation between the pointing and knuckle localisation task in terms of finger length, there was no correlation between these tasks in terms of hand width, *r*(18) = 0.020, n.s.

### Discussion

This experiment provided clear replications of the distortions previously reported for the pointing (e.g., Longo and Haggard, 2010, 2012a; Longo, 2014, 2015d; Mattioni and Longo, 2014), line length (Longo and Haggard, 2012b), and knuckle localisation (Longo, 2015a; Margolis and Longo, 2015) tasks. While the direction of these distortions was in the same direction in all three tasks (underestimation of finger length, overestimation of hand width), they were dramatically different in magnitude. Indeed, underestimation of finger length in the knuckle localisation task was less than a third (29.2%) of that in the pointing task. There was, however, a strong correlation between underestimation of finger length in the pointing and knuckle localisation tasks, suggesting that conceptual distortions do make a contribution to this distortion. In the case of the overestimation of hand width, distortion in the knuckle localisation task was an order of magnitude smaller (8.7%) than that in the pointing task.

## Experiment 2

The results of Experiment 1 suggest that both perceptual and conceptual factors contribute to the distorted representations found in the pointing task. Nevertheless, the tasks used in Experiment 1 differ in many ways, making direct comparison of the magnitude of effects across tasks difficult. The goal of this experiment was to more directly investigate the role of perceptual and conceptual distortions using the same task. We compared distortions of perceptual hand maps in a standard version of the pointing task in which the participant made proprioceptive judgments of the location of landmarks of their own hand to those in a condition in which they made judgments from memory about the location of the same landmarks on a prosthetic rubber hand which they saw before each block. Perceptual distortions of hand structure should be specific to the participant’s own hand, whereas conceptual distortions should apply to hands generally and thus appear in both conditions. Critically, while the conditions differ in terms of whether judgments are based on immediate proprioceptive signals or on visual memory, the task and manner of responding was identical in both cases.

### Methods

#### Participants

Twenty healthy individuals (11 female) between 19 and 73 years of age participated (*M*: 29.7 years). None had participated in Experiment 1. All but two were right-handed as assessed by the Edinburgh Inventory (*M*: 63.56; range: -100 to +100).

#### Procedure

Procedures in the *Own Hand* condition were similar to the pointing task in Experiment 1. In the *Rubber Hand* condition, a prosthetic left hand was placed on the table in approximately the same location and posture as the participant’s own hand in the other condition. At the start of each rubber hand block, the participant was asked to look at the rubber hand for 10 s before it was covered. Responses were then made as in the own hand condition except that they were based on the participant’s visual memory of the location of the rubber hand. During the rubber hand condition, the participant’s left hand rested in their lap.

There were four blocks of 30 trials each, two for the *Own Hand* and two for the *Rubber Hand* condition. The two conditions were counterbalanced across the four blocks in ABBA fashion, the first condition being counterbalanced across participants.

### Results

**FIGURE 5:**
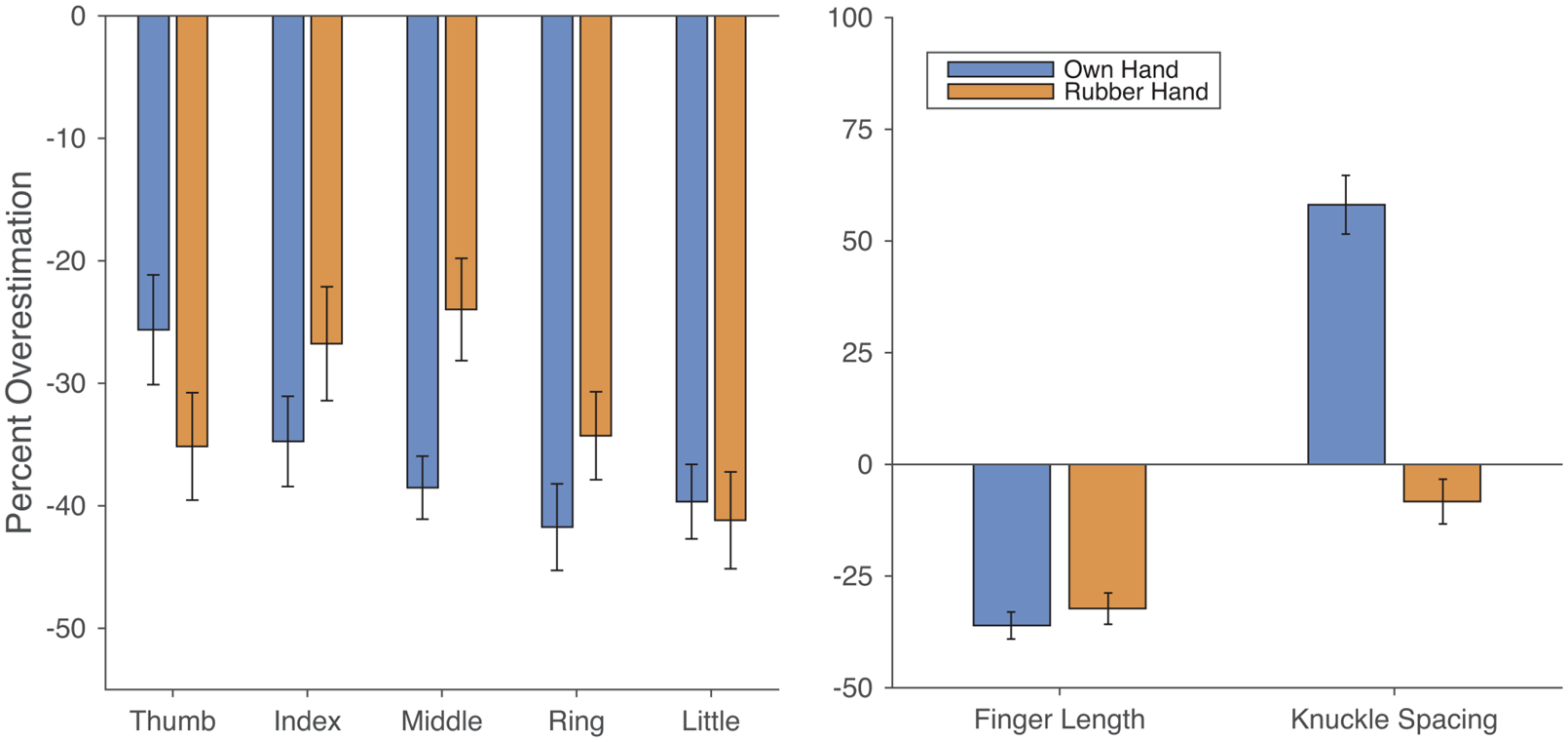
**Results from Experiment 2.** **(Left)** Similar underestimation of finger length was found in both the Own Hand and Rubber Hand conditions. **(Right)** In contrast to the similar underestimation of finger length, overestimation of hand width was found only in the Own Hand condition.

**FIGURE 6:**
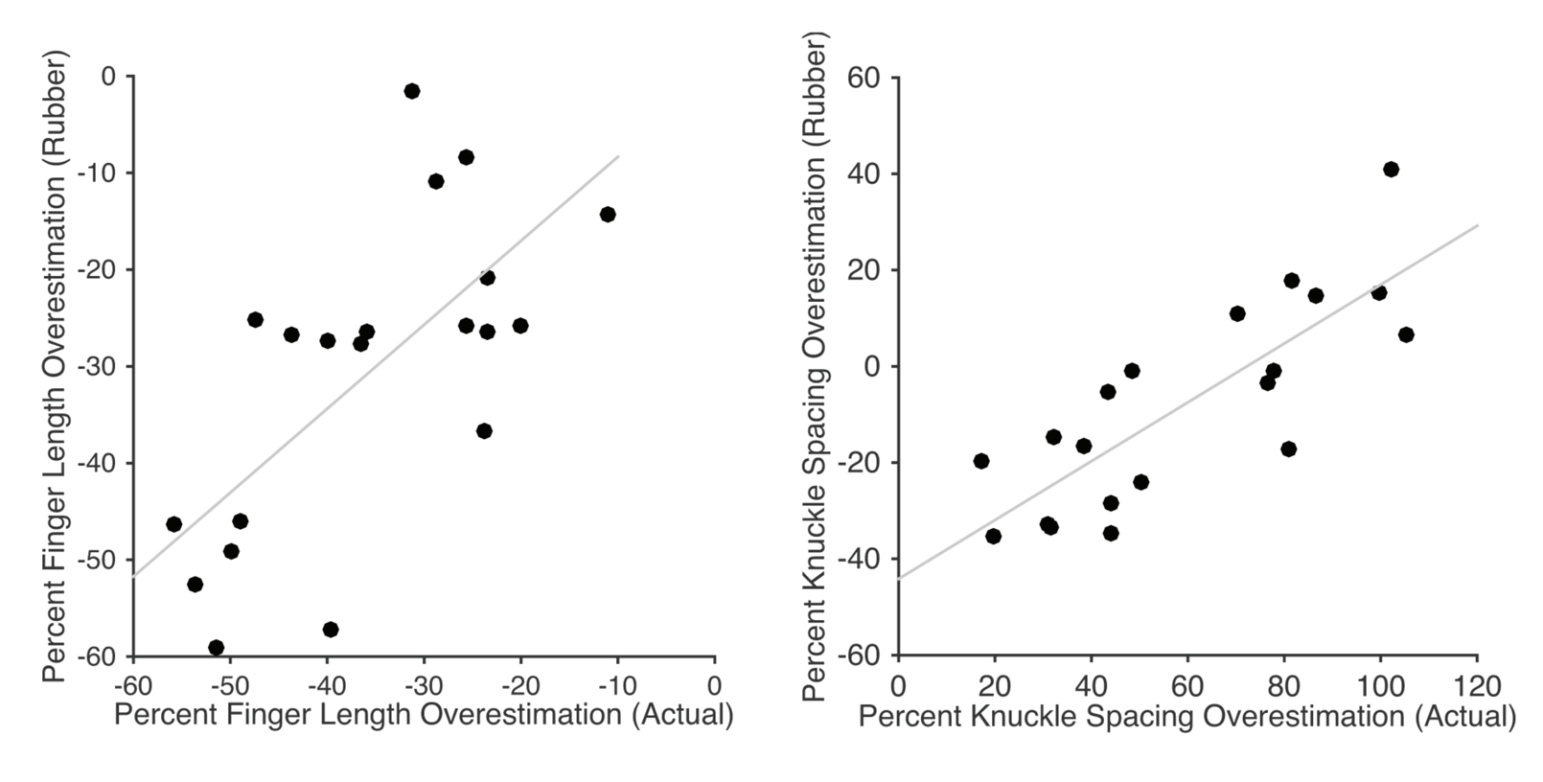
**Scatterplots showing relations between underestimation of finger length **(left)** and overestimation of hand width **(right)** for the participant’s own hand and the rubber hand**.

The results are shown in **Figure 5**. Across fingers there was clear underestimation of finger length both for the participant’s own hand (*M*: 36.1%), *t*(19) = 11.93, *p* < 0.0001, *d* = 0.98, and the rubber hand (*M*: 32.3%), *t*(19) = 9.21, *p* < 0.0001, *d* = 1.97. The magnitude of underestimation did not differ significantly between the two hands, *t*(19) = 1.51, *p* = 0.148, *d*_z_ = 0.314. There was a clear correlation across participants between the magnitude of underestimation on the two hands, *r*(18) = 0.682, *p* < 0.001 (see **Figure 6**, left panel). For the own hand condition, there was a clear radial-ulnar gradient in the magnitude of underestimation across the five fingers, (mean β = -3.50%/finger), *t*(19) = -4.20, *p* < 0.0005, *d* = 0.976. This effect did not quite reach significance for the rubber hand (mean β = -1.96%/finger), *t*(19) = -1.98, *p* = 0.0623, *d* = 0.46, though the difference in slope between the two conditions was not significant, *t*(19) = 1.41, *p* = 0.175, *d*_z_ = 0.324.

In contrast to the similar underestimation of finger length, there were striking differences between the conditions in overestimation of hand width. For the own hand condition, there was clear overestimation of hand width (*M*: 58.12%), *t*(19) = 8.85, *p* < 0.0001, *d* = 2.06. For the rubber hand condition, however, there was a trend in the opposite direction (*M*: -8.32%), *t*(19) = -1.66, *p* = 0.11, *d* = 0.39. There was a clearly significant difference between the magnitude of overestimation in the two conditions, *t*(19) = 17.31, *p* < 0.0001, *d*_z_ = 4.01. Despite the fact that the direction of distortion went in opposite directions in the two conditions, there was nevertheless a significant positive correlation between them, *r*(18) = 0.811, *p* < 0.0001 (see **Figure 6**, right panel).

### Discussion

The results from this experiment show a striking dissociation between different distortions. Underestimation of finger length was highly similar for the participant’s own hand and the rubber hand. This result is consistent with the interpretation of this underestimation as a conceptual distortion, generic to the representation of hands in general, and not specific to the perceptual representation of the participant’s own hand. In striking contrast, the overestimation of hand width was found only for the participant’s own hand, with a slight trend in the opposite direction found for the rubber hand. This suggests that this overestimation reflects a perceptual distortion of the representation of the participant’s own hand.

## General Discussion

Together, these results demonstrate the contribution of both perceptual and conceptual factors to the distortions of hand representation underlying position sense. In Experiment 1, we replicated the recent finding that participant’s overtly judge their knuckles as lying farther forward in their hand than they really are (Longo, 2015a; Margolis and Longo, 2015), and further showed that this bias predicts the magnitude of underestimation of finger length in the pointing task. While the magnitude of knuckle mislocalisation was only big enough to account for less than one third of the underestimation of finger length, this result reveals a clear contribution of conceptual factors to this distortion. In contrast, the localisation of knuckles within the hand was completely unable to account for the overestimation of hand width in the pointing task, suggesting that this reflects a perceptual distortion of hand shape. The results from Experiment 2 were consistent with those of Experiment 1. Participants showed similar underestimation of finger length when localizing landmarks on their own hand using position sense and on a prosthetic hand using visual memory, consistent with the idea that this reflects a conceptual distortion of how people represent hands in general. In contrast, overestimation of hand width was highly specific to the participant’s own hand, suggesting that it reflects a perceptual distortion of the participant’s representation of their own body.

These results demonstrate that the distortions that we and others have previously described are unlikely to arise from a single cause. Recent studies have demonstrated distorted body representations on an increasingly wide range of tasks based on, for example, proprioception (e.g., Longo and Haggard, 2010; Longo, 2014; this study), tactile distance perception (e.g., Taylor-Clarke et al., 2004; Longo and Haggard, 2011; Longo et al., 2015a), tactile localisation (e.g., Mancini et al., 2011; Margolis and Longo, 2015), overt size estimates of body parts (e.g., Linkenauger et al., 2009, 2015; Longo and Haggard, 2012b; this study), localisation of landmarks within the body (e.g., Longo, 2015a; Margolis and Longo, 2015; this study), and judgments of overall body configuration (e.g., Fuentes et al., 2013a,b,c). Given such a list, it should perhaps not be surprising that more than one factor is responsible for generating these distortions. The present results contribute to understanding such effects by revealing two specific types of distortion which underlie body representation in the specific case of position sense. These findings provide further evidence for the interpretation that, far from being a certain signal of pathology, distorted body representations are a ubiquitous aspect of healthy cognition.

While we have argued to this point for a largely categorical distinction between perceptual and conceptual distortions, it is worth speculating on the possible relations between them. In Experiment 1, for example, there was a clear progressive reduction in the magnitude of finger length underestimation from the pointing to the line length to the knuckle localisation task. It is important to understand what causes both the similarities and differences among these tasks. One possibility is that this bias results from some basic distortion of somatosensory cortical organization which is progressively corrected at sequential stages of processing. On this interpretation, different representations of the body are not categorically distinct, but reflect a hierarchical organization based on sequential stages of perceptual processing (cf. Longo, 2015b). Longo and Haggard (2012b), for example, suggested that the distortions seen in the pointing and line length tasks might reflect different weighted combinations of (distorted) somatosensory representations and (largely veridical) visual representations. The similar biases found in the two tasks would then arise from both tasks inheriting distortions of somatosensory cortical maps, while the smaller magnitude of distortions in the line length task would reflect the reduced influence given to somatosensory representations for overt judgments of body size than for position sense. Historically, research on body representations has focused on identifying dissociations between putatively distinct body representations (e.g., Sirigu et al., 1991; Gallagher and Cole, 1995; Paillard, 1999; Schwoebel and Coslett, 2005). The present results emphasize the importance of understanding not only the distinctions between, but also the relations among representations of the body used for different tasks.

The present results are also relevant for a recent interpretation of distortions put forward by Saulton et al. (2015). These authors suggested that the distortions of hand shape described by Longo and Haggard (2010) could result from a general bias to misrepresent the shape of elongated shapes. They reported finding biases analogous to (but smaller in magnitude than) those described by Longo and Haggard (2010) when participants localized landmarks on non-hand shapes through visual memory. Experiment 2 in the present study provides a strong test of this idea. If the distortions for the participant’s own hand are a reflection of representation of objects with that general shape, the rubber hand (being very similar in shape to participants’ hand) should have shown very similar distortions. While similar distortions were found for underestimation of finger length, no overestimation of hand width was found for the rubber hand. This result provides strong evidence that such overestimation does not result merely from the fact that the hand has an elongated shape, as suggested by Saulton et al. (2015).

These results fit with previous results demonstrating that the specific type of stimuli or judgment required of participants in body representation tasks has important effects on the type and magnitude of distortion observed. For example, meta-analyses of studies on patients with eating disorders have found that ‘depictive’ tasks which involve an image of the overall form of the body show larger (Cash and Deagle, 1997) and more stable (Smeets et al., 1997) distortions than ‘metric’ tasks which involve only estimates of body-part size. Recent studies with healthy participants have found distortions which differ either qualitatively (e.g., Longo and Haggard, 2010, 2012b) or quantitatively in terms of their magnitude (Longo and Haggard, 2012b) depending on the specific nature of the task employed. The exact stimulus and task dimensions which drive these differences remain poorly understood. Determining the factors which modulate the presence and magnitude of distorted body representations is an important goal for future research.

The present results may also have implications for understanding disruptions of body representations in clinical disorders. For example, in the literature on eating disorders there has been debate about whether distortions of perceived body size in patients with conditions such as anorexia nervosa reflect a genuine perceptual distortion of the body image, or rather reflect negative attitudes toward the body (e.g., Ben-Tovim et al., 1990; Cash and Deagle, 1997). The present distinction between perceptual and conceptual distortions has some similarity to that distinction, although there is no obvious link between what we have called conceptual distortions and affective responses. Understanding the connections between the distortions we report in healthy people and those seen specifically in patients with eating disorders is an important area for future research. Traditionally, disrupted body image in eating disorders has been linked to the visual depiction of bodies in Western media (Becker and Hamburg, 1996; Derenne and Beresin, 2006). In contrast to this view, recent neuroimaging results have suggested that patients with eating disorders may have reduced activation (Uher et al., 2005), gray-matter density (Suchan et al., 2010), and functional connectivity (Favaro et al., 2012; Suchan et al., 2013) in areas of the ventral visual cortex involved in visual perception of bodies. Such results suggest that, seemingly paradoxically, individuals with eating disorders may actually be less reliant on visual perception of bodies than healthy individuals. This raises the possibility that one contribution to body image disturbances in eating disorders might be that somatosensory distortions, which remain implicit in healthy cognition, may rise to conscious awareness, leading to analogous distortions in the subjective body image (cf. Longo, 2015b).

## Conclusion

This study suggests that both perceptual and conceptual factors contribute to the distorted body representations that appear to underlie human position sense. Such distortions may not reflect a single phenomenon with a single underlying cause. It will be critical in future research to develop a more complete understanding of the different factors which produce distortions of body representations, how these factors are related and interact as well as how they manifest themselves in different tasks.

## Conflict of Interest Statement

The authors declare that the research was conducted in the absence of any commercial or financial relationships that could be construed as a potential conflict of interest. The Associate Editor Christoph Braun declares that, despite being affiliated to the same institution as author Stefania Mattioni, the review process was handled objectively and no conflict of interest exists.
